# Point-of-Care Diagnostics in Coagulation Management

**DOI:** 10.3390/s20154254

**Published:** 2020-07-30

**Authors:** Sebastian D. Sahli, Julian Rössler, David W. Tscholl, Jan-Dirk Studt, Donat R. Spahn, Alexander Kaserer

**Affiliations:** 1 Institute of Anesthesiology, University and University Hospital Zurich, 8091 Zurich, Switzerland; sebastian.sahli@usz.ch (S.D.S.); julian.roessler@usz.ch (J.R.); david.tscholl@usz.ch (D.W.T.); donat.spahn@usz.ch (D.R.S.); 2 Division of Hematology, University and University Hospital Zurich, 8091 Zurich, Switzerland; jan-dirk.studt@usz.ch

**Keywords:** hemorrhage, coagulation management, ROTEM^®^, platelet function test, point-of-care systems

## Abstract

This review provides a comprehensive and up-to-date overview of point-of-care (POC) devices most commonly used for coagulation analyses in the acute settings. Fast and reliable assessment of hemostasis is essential for the management of trauma and other bleeding patients. Routine coagulation assays are not designed to visualize the process of clot formation, and their results are obtained only after 30–90 m due to the requirements of sample preparation and the analytical process. POC devices such as viscoelastic coagulation tests, platelet function tests, blood gas analysis and other coagulometers provide new options for the assessment of hemostasis, and are important tools for an individualized, goal-directed, and factor-based substitution therapy. We give a detailed overview of the related tests, their characteristics and clinical implications. This review emphasizes the evident advantages of the speed and predictive power of POC clot measurement in the context of a goal-directed and algorithm-based therapy to improve the patient’s outcome. Interpretation of viscoelastic tests is facilitated by a new visualization technology.

## 1. Introduction

Severe bleeding, e.g., after trauma or during surgery, requires adequate coagulation management [1]. For this purpose, point-of-care (POC) tests allow a fast assessment of the hemostasis, and provide important guidance when a coagulation algorithm is used [2,3]. If bleeding is aggravated by coagulopathy, the negative impact on survival is significant [4]. A factor-based coagulation management guided by POC diagnostics is therefore considered the gold standard in hemostatic resuscitation [1]; it leads to a decreased transfusion requirement and improved outcome in patients with major trauma [2,5], undergoing cardiac surgery [6,7] or suffering from postpartum hemorrhage [8,9].

This review provides a comprehensive and up-to-date overview of point-of-care (POC) devices most commonly used for coagulation analyses in the acute settings. POC testing refers to laboratory analyses that are performed at the site where the patient is treated. We divide the POC assays for coagulation management into three major groups: (1) combined assessment of clotting time and clot quality and stability (viscoelastic methods); (2) analyzers of the platelet function and (3) analyzers of the plasmatic coagulation (coagulometers). In addition, blood gas analyzers are addressed because they provide important physiological parameters. For a correct application, a detailed knowledge of the assay’s functional characteristics and limitations is required. In addition to this, we will also focus on the important issue of their clinical application.

While fundamental physiological parameters like acidosis, electrolyte shifts or anemia can be evaluated instantly in the extended blood gas analysis, the assessment of hemostasis is more challenging. Standard coagulation assays such as prothrombin time (PT), activated partial prothrombin time (aPTT) or thrombin time (TT) are not designed to visualize the process of clot formation or clot stability [3]. Moreover, because of the processes of sample preparation and centrifugation, analysis and validation of the results standard laboratory results are available only after 30–90 m [10] or even later. They are therefore of limited value for coagulation management in the initial acute phase, especially if repetitive analyses are needed [11]. A faster alternative is POC assays, which include viscoelastic coagulation tests, platelet function tests and coagulometers. They allow the assessment of clot formation, clot stability and clot lysis in real-time, also to evaluate for a possible intake of oral anticoagulants or platelet inhibitors. This review cannot present all of the available POC devices, and focuses on the most widely available and commonly used tests.

## 2. Standard Laboratory Coagulation Tests

**Figure 1:**
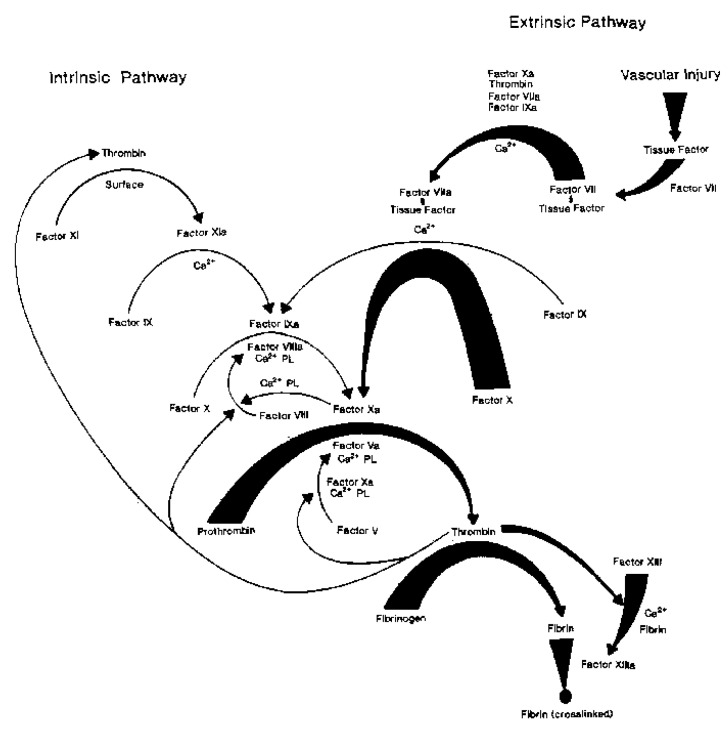
Coagulation cascade and fibrin formation by the intrinsic and extrinsic pathways. The heavy arrows show the extrinsic pathway, triggered by vascular injury and the expression of tissue factor. On the left side, by light arrows, the intrinsic pathway is shown. In the common pathway, thrombin is generated, which converts fibrinogen to fibrin (heavy arrows, too). Reprinted with permission from [13]. Copyright© 1991 American Chemical Society, Washington, DC, USA.

The bleeding time was first used to assess the global hemostasis in vivo [12]. Being an invasive test, its reproducibility is poor and it was therefore abandoned. A model of the plasmatic coagulation (see Figure 1) distinguishes an extrinsic pathway, which starts with tissue factor/factor VII activation, an intrinsic pathway that is activated by contact factors, and a common pathway comprising the coagulation factors II, V and X [13]. The coagulation cascade is a tightly controlled series of enzymatic and cell-based reactions, designed to generate thrombin, which converts fibrinogen to fibrin, and requires co-factors such as phospholipids and calcium ions. It is usually subdivided into an initiation phase, an amplification phase and a propagation phase [14].

Global tests of coagulation are the prothrombin time (PT), which reflects the extrinsic pathway of the coagulation cascade, and the activated partial thromboplastin time (aPTT), which reflects the intrinsic pathway. PT, described first by Quick in 1935 [15], is still referred as “Quick’s PT” or “Quick value”. Originally developed to determine PT (factor II of the coagulation cascade), it is now clear that it depends especially on factor VII together with the factors X, V, II and fibrinogen. Changes over time have made the test more specific for the vitamin K dependent clotting factors (VII, X, V and II). The initiation of clotting, specifically of the intrinsic pathway, is naturally triggered by negatively charged phospholipid surfaces (platelet effect). This reaction is artificially replaced by contact phase activators. In the past through cephalin, currently through minerals as kaolin or celite and more rarely silica or glass dust. The aPTT depends initially on factor XII and XI and reflects the intrinsic pathway, especially factors VIII, IX and XI. Originally designed in 1953 by Langdell et al. [16] as a simple one-stage test for hemophilia. At that time the test was activated by cephalin (phospholipid) and consequently replaced by kaolin (mineral) to optimize the contact phase [17]. Both tests, PT and aPTT, reflect the common pathway of factors II, X and XIII. The thrombin time (TT) covers the final step of fibrin polymerization. These tests are however performed in a standardized and artificial setting. They reflect the situation in vivo only partially, and may be influenced by a variety of preanalytical factors. Similarly, fibrinogen according to Clauss [18], which is a modification of TT and the most commonly used assay method may overestimate the fibrinogen concentration in the presence of hydroxyethyl starch (but not gelatin) [19].

## 3. Viscoelastic Tests

Viscoelastic testing was first described by Hartert in 1948 [20], and was established in the following years. Initially, a non-activated clot measurement was performed, which led to variability and longer measurement times. The test was also susceptible to vibration, which limited the use of viscoelastic tests. Today, the tests are initiated with citrated whole blood and defined activators or inhibitors. The thrombelastographic system (TEG^®^) is in use more often in America, and the rotational thrombelastic system (ROTEM^®^) in Europe. Different from standard coagulation assays, viscoelastic methods display clot formation and clot stability in real time. They permit detection of a delayed initiation of coagulation, a reduced fibrinogen level, an increased fibrinolytic activity and of the platelets’ contribution in whole blood. Viscoelastic assays are fast and give first results within 5–10 m. In Section 3.4 we focused on the clinical impact of these early parameters. Viscoelastic tests are of an advantage in acute situations such as trauma-induced coagulopathy, transfusions management, intra- and postoperative bleeding and targeted hemostatic therapy. Using guidance by viscoelastic tests, superior outcomes in trauma patients [5,11], cardiac surgery [6,7] and postpartum hemorrhage [8,9] are proved. Further, they allow the detection of a delayed coagulation initiation, diminished fibrinogen level, an increased fibrinolytic activity and of the platelet level in whole blood [21]. They may also indicate the presence of anticoagulants, and give additional information in patients with hypercoagulability [22] or substitution therapy in hemophiliacs [23]. Commonly used viscoelastic assays and their clinical applications are described in the next chapters.

### 3.1. Rotational Thrombelastometry-ROTEM^®^

**Figure 2:**
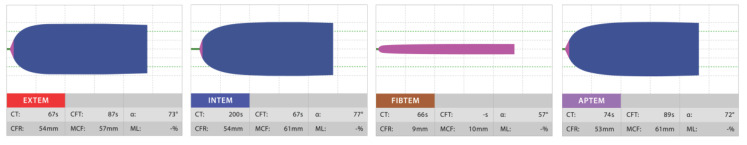
Picture of the ROTEM^®^ temogram displaying the four channels **EXTEM**, **INTEM**, **FIBTEM** and **APTEM**. Reprinted with the kind permission from the Instrumentation Laboratory, Bedford, MA, USA.

Rotational thrombelastometry (ROTEM^®^, Instrumentation Laboratory, Bedford, MA, USA) displays the processes of clot formation and subsequent clot lysis in a direct graphical manner. 300 µL of citrated whole blood is incubated at 37 °C in a cup using a software-controlled and self-guided pipette. The cuvette is fixed, and a pin is inserted into the blood and rotates alternately with an angle of 4°75’ around its longitudinal axis. Recalcification activates the clotting in the cup. Different reagents are added to classify the hemostasis defect. A signal is generated, and translated from the pin via an optical detector into a visual graph [22]. The ROTEM^®^ delta system provides four channels for parallel testing different aspects of the patient’s hemostasis (see Figure 2). The new ROTEM^®^ sigma operates by the same principles. It is automated with ready-to-use cartridges for simultaneous testing.

Compared with a monoanalysis, the simultaneous assessment of different assays allows a more comprehensive diagnosis [24]. These assays are described in detail and summarized in Table 1: For **EXTEM**, tissue thromboplastin is added to activate the extrinsic pathway of coagulation. The reaction depends on the activity of coagulation factors X, VII, V, II, fibrinogen and platelets. For **INTEM**, ellagic acid and phospholipids are added as contact phase activators. Beside the coagulation factors XII, XI, IX, VIII, X, V and II, clot firmness reflects both platelet and fibrin contribution [25]. INTEM reflects the intrinsic coagulation pathway. **FIBTEM** is an EXTEM-based assay. Since it contains a potent platelet inhibitor (cytochalasin D), clot formation depends mostly on fibrinogen concentration and fibrin polymerization. Combining FIBTEM and EXTEM permits distinction of thrombocytopenia and hypofibrinogenemia [26]. **APTEM** is also an EXTEM-based assay using the addition of an antifibrinolytic (earlier aprotinin, now tranexamic acid) to simulate an antifibrinolytic therapy. HEPTEM is an INTEM based assay with the same contact phase activator, and heparinase is added to discriminate between a heparin effect and a high dose of protamine [27]. Additional assays are available, but are considered less clinically relevant (and therefore used only occasionally or for research purposes). ECATEM uses ecarin as an activator, a viper venom with a thrombin-like effect; this clotting time is altered by direct thrombin inhibitors such as argatroban, bivalirudin or dabigatran but not by heparin. At present, ECATEM is distributed only in Europe [28]. NATEM does not use a specific activator and the coagulation process is only started by recalcification. It is sensitive to endogenous activators of coagulation, such as a tissue factor expressed on circulating monocytes. It may help to detect pathophysiological changes in trauma-induced coagulopathy and coagulation alterations in sepsis [29].

**Figure 3:**
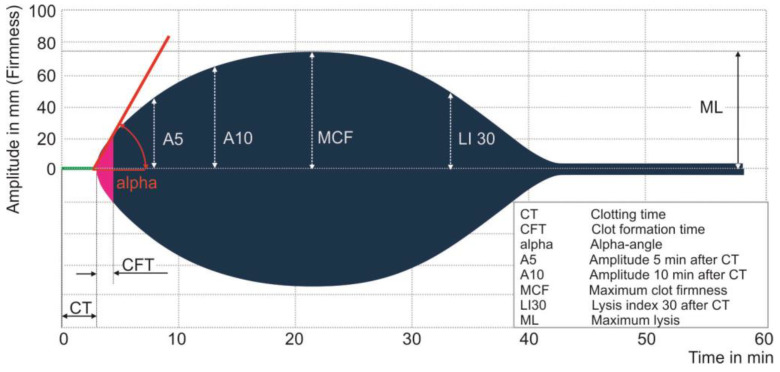
Picture of ROTEM^®^ temogram illustrating the parameters characterizing clot formation. Reprinted with the kind permission from the Instrumentation Laboratory, Bedford, MA, USA.

A set of parameters (see Figure 3) was used to characterize clot formation, clot firmness and clot lysis [30]. These were compared with TEG^®^ parameters in the next section. During coagulation activation, the clotting time (CT) is defined as the time from the beginning of the test until a clot firmness amplitude of 2 mm was achieved, and reflected the velocity of thrombin generation. The clot formation time (CFT) indicates the time between a clot amplitude of 2 and 20 mm and reflects the kinetics of clot formation. The alpha angle (α) is the tangential angle of the curve between 0 and 20 mm. Clot firmness is described by the maximum clot firmness (MCF), which is the maximal clot amplitude reached and reflects the mechanical strength of the clot. Quickly available parameters are the amplitude of clot firmness 5, 10 or 20 m after CT (A5, A10 and A20, respectively). A5 and A10 correlate especially well with MCF, providing fast and reliable POC information [31]. Finally, clot lysis is defined by the maximum lysis (ML) as the delta between MCF and the lowest amplitude following MCF. Likewise, the percentage of MCF (lysis index LI), which is present 30 or 60 m after CT (LI30 and LI60, respectively), provide faster information [32].

### 3.2. Visual Clot

**Figure 4:**
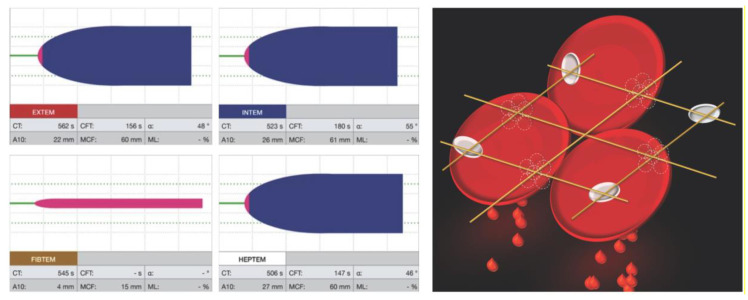
Coagulation factor deficiency: left, displayed by a four channel ROTEM^®^ report (EXTEM, INTEM, FIBTEM and APTEM); right, by Visual Clot visualization technology.

**Figure 5:**
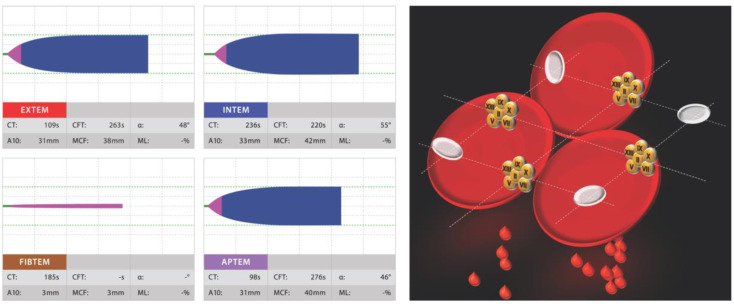
Low fibrinogen level: left, displayed by a four channel ROTEM^®^ report (EXTEM, INTEM, FIBTEM and APTEM); right, by Visual Clot visualization technology.

Hospitals around the world are using viscoelastic tests for POC coagulation assessment. The way their results are displayed is however not self-explanatory, and their interpretation may be challenging. Visual Clot is a situation awareness-oriented visualization technology for thrombelastometric results [33]. Examples are shown in Figure 4 and Figure 5. An algorithm transforms parameters of rotational thrombelastometry into an animated model of the blood clot under investigation. In a prospective dual-center study [33], 60 physicians interpreted results assisted by Visual Clot vs. standard procedures, and based therapeutic decisions on their interpretation. Visual Clot resulted in an overall of 100% correct decisions vs. 44% for standard procedures. The perceived cognitive workload using Visual Clot was less, and diagnostic confidence was rated higher. Additionally, the correct interpretation of Visual Clot did not depend on previous knowledge and experience with rotational thrombelastometry. This new technology resulted in a faster and more accurate detection of alterations, and a higher rate of correct therapeutic decisions in simulated cases [33]. In a mixed qualitative/quantitative study, 92% percent of 42 physicians indicated a preference to have Visual Clot results displayed in addition to the standard result, and most described it as intuitive and easy to learn [34]. This indicates a potential benefit by the situation awareness-oriented presentation of information, taking into account the capabilities and requirements of human operators. Visual clot was developed by the same research group at the University of Zurich as Visual Patient^®^, and both rely on the same graphic principles (details can be found in a recent review article published in *Sensors*) [35].

An additional educational video was provided on Visual Clot with different clinical scenarios (e.g., bleeding, hyperfibrinolysis and heparin effect): https://1drv.ms/v/s!AjkumfX_cNxMyiaZ9jV39Cvn1_rD?e=4TD7OO.

### 3.3. Thrombelastography—TEG^®^

Thrombelastography (TEG^®^, Haemonetics Corp., Boston, MA, USA) provides information similar to the ROTEM^®^, but uses a different technique. While ROTEM^®^ uses a rotating pin, the TEG^®^ 5000 uses a cup oscillating by an angle of 4°45’ around the pin. A torsion wire translates the movement into a signal and ultimately into a graph. The test uses citrated whole blood at 37 °C. The formation of fibrin filaments between the cuvette wall of and the pin after recalcification and activation inhibits the cup’s movement, which is recorded as a curve over time. The new TEG^®^ 6 s system detects the same physical properties of clot viscoelasticity as the TEG^®^ 5000 but uses a resonance method. The sample is exposed to vibration at a fixed frequency, which is detected by LED [36]. This test is fully automated and uses prefabricated microfluid cartridges for simultaneous testing. No pipetting is required.

Four main TEG^®^ assays and one native assay were used. An overview is given in Table 2. A combination of these assays is important as well. RapidTEG™ assay contains a tissue factor together with kaolin as activators. Thereby, both the intrinsic and the extrinsic pathway are triggered, similarly to the activated clotting time (ACT). Kaolin assay initiates the contact activation of coagulation and reflects the intrinsic coagulation pathway. Clot firmness reflects the coagulation factors XII, XI, IX, VIII, X, V and II, also platelet and fibrin contribution. The functional fibrinogen assay contains tissue factor for activation together with abciximab, a potent inhibitor of the platelet fibrinogen receptor GPIIb-IIIa, thereby eliminating the contribution of platelets. In comparison with the kaolin assay, it permits a qualitative statement of the contribution of fibrinogen concentration and fibrin polymerization to clot formation. HTEG assay is based on the RapidTEG™ assay, additionally containing heparinase to neutralize an effect of unfractionated heparin. The Native assay only recalcifies whole blood, generating a long R time [37].

The initiation of coagulation is described by the reaction time (R value as the time between the starting of the test and the beginning of clot formation). The K value then describes the kinetics of clot formation, between the end of R until the clot reaches 20 mm. The widest vertical dimension of the graph defined as maximum amplitude (MA) represents the clot strength. Clot lysis (CL) is described by the percentage of the clot lysed after 30 and 60 m (CL30 and CL60).

As presented in Table 3, ROTEM^®^ and TEG^®^ parameters were comparable but not interchangeable. Ziegler et al. showed a high degree of correlation of ROTEM^®^ and TEG^®^ parameters using their most recent versions [38]; however, device-specific algorithms for interpretation of the results are mandatory.

### 3.4. Early Viscoelastic Variables to Predict Transfusion and Mortality

Bleeding is a leading cause of morbidity and mortality in trauma patients [4,39]. Hemorrhagic death within the first 24 h following trauma was 94%, as described in a prospective multicenter study conducted by Holcomb et al. [40]. Interestingly, 60% of these bleeding patients died within the first 3 h after hospitalization. Consequently, rapid and targeted intervention is essential for the survival of bleeding patients.

The prospective study by Hagemo et al. [41] confirmed the ROTEM^®^ early parameter A5 in EXTEM and FIMBTEM assays as a marker for acute traumatic coagulopathy and predictor for massive transfusion. The systematic review by Veigas et al. 2016 [32] supports the evidence that A5 and A10 in EXTEM and FIBTEM assays diagnose coagulopathy earlier and predict blood transfusion and mortality. In addition, an abnormal early clot lysis index LI30 associates with the presence of fibrinolysis.

Additionally, for TEG^®^ assays, a strong correlation of early amplitudes (A5 and A10) in the rTEG, kTEG and TEG FF was confirmed [42]. These early parameters A5 and A10 were significantly lower in transfused patients in rTEG and TEG FF assays (1–9 units of red blood cells (RBC) as well as in the kTEG assay too, if more than 10 RBC were transfused. Even significant predictors of mortality were found in relation to the early amplitudes in the A10 kTEG and A5 TEG FF assays. Comparable data on early amplitudes were already shown 2014 by Meyer et al. [43].

### 3.5. ClotPro^®^

The viscoelastic analyzer ClotPro^®^ (Haemonetics Corporation, Boston, MA, USA; formerly enicor GmbH, Munich, Germany) provides six channels for parallel testing. It has a unique Active-Tip™ technology with the dried reagents contained in a sponge at the pipette tip. When a blood sample is pipetted the reagents dissolve and activate the sample. Thereby, manual handling of liquid reagents is obsolete. For testing, 340 µL of citrated whole blood is pipetted into a cylindrical cup with an immersed pin. The cup rotates and the pin is fixed. Following activation blood adheres to the surfaces of cup and pin, and the strength of the clot is continuously detected and displayed graphically. Established parameters of thrombelastography (CT, CFT, A5, A10, MCF, ML, LT, α and CLI) are used. At present, ClotPro^®^ is not commercially available in the USA at current times [44].

It provides nine types of assays: for screening, EX-test, FIB-test, AP-test, IN-test and HI-test. New assays for drug monitoring are the RVV-test, ECA-test, TPA-test and NA-test. The EX-test assesses the extrinsic coagulation pathway and its interaction with platelets. A recalcified sample is activated with a tissue factor, and hexadimethrin bromide is added to neutralize heparin. The FIB-test determines the fibrinogen level and fibrin polymerization. The recalcified sample is again activated with the tissue factor, and platelets are inhibited by cytochalasin D and a GPIIb-IIIa antagonist, too. Hexadimethrin bromide is also added. The AP-test permits evaluation of the extrinsic coagulation pathway in a fibrinolysis-independent manner by adding a plasmin antagonist (aprotinin). The IN-test evaluates the intrinsic coagulation pathway and its interaction with platelets. The recalcified sample is activated by ellagic acid. It is sensitive to heparin and FVIII. The HI-test (IN-test based) offers evaluation of the intrinsic coagulation pathway insensitive to heparin by the addition of heparinase. There are new assays designed for drug monitoring: the RVV-test is sensitive to factor Xa-antagonists by using Russel Viper Venom, an activator of factor X. The ECA-test uses the prothrombin-activating viper venom ecarin. It detects thrombin inhibitors such as dabigatran. The TPA-test uses recombinant tissue plasminogen activator (r-tPA for the detection of antifibrinolytics. Finally, the NA-test assesses non-activated coagulation.

### 3.6. Sonoclot^®^

Another viscoelastic analyzer is Sonoclot^®^ (Sienco Inc., Boulder, CO, USA). This test was described in 1975 by Kaulla [45]. It was further developed and uses whole blood. At the beginning, a hollow disposable plastic is attached to an ultrasonic transducer. The blood sample is pipetted into a container with coagulation activators or inhibitors. The transducer is then inserted at a defined height and the sample is oscillated at 200 Hz with a vertical deflection of 1 µm. There are different analyzers available, the one-channel SC1 and the two/four-channel SCP2/SCP4. Additional tests are available [46]: The kACT Kit as a kaolin-activated clotting time intended for high dose heparin management providing quantitative ACT and clot rate results. The SonACT Kit is celite-activated and intended for high dose heparin management, too. The gbACT Kit is a glass bead activated clotting time, which is well designed for high dose heparin management. The gbACT+ Kit is glass bead activated and designed for use in non-heparinized patients. The H-gbACT+ Kit is glass bead activated and contains heparinase, intended for standard clotting assessment patients receiving heparin. The aiACT Kit uses celite and other minerals for contact activation, and is intended for high dose heparin anticoagulation management. A non-activated Kit is available, too. Parameters for initial fibrin formation (SonACT), clot rate (CR), maximum clot strength (peak amplitude and time to peak) and fibrinolysis (R3) are transmitted [47].

## 4. Point-of-Care Guided Therapy

Targeted hemostasis management of trauma patients guided by POC diagnostics is defined as the gold standard in resuscitation [1]. At least one in four trauma patients suffers from trauma-induced coagulopathy upon hospital admission [48,49]. POC guided hemostatic resuscitation with target guided coagulation factor therapy showed a higher probability of survival [5,50] and reduced transfusion of allogeneic blood products [2,11,42]. Furthermore, empiric administration of fresh frozen plasma (FFP) induces dilution coagulopathy and hypofibrinogenemia [51]. Transfusion itself is associated with increased mortality [52] and a high risk of adverse events such as lung damage, volume overload and heart failure, kidney damage, transmission of infections and immunological activation [53,54]. Besides trauma, pathological changes can be caused through intra- and postoperative bleeding.

### 4.1. Viscoelastic Parameters for POC Guided Therapy

Using ROTEM^®^ assays, Theusinger et al. [21] showed in a retrospective study that the parameters CFT, α-angle and MCF in EXTEM, INTEM and APTEM are significantly (*p* ≤ 0.003) associated with fibrinogen and platelet levels, and FIBTEM MCF parameter significantly (*p* ≤ 0.003) with fibrinogen. A large retrospective study by Chow et al. [55] proved through TEG^®^ assays that the kTEG parameter MA best diagnoses hypofibrinogenemia (fibrinogen < 200 mg/dL, *p* < 0.001) and accurately diagnoses all parameters (MA, k-time, and alpha-angle; *p* < 0.001) of severe hypofibrinogenemia (fibrinogen < 100 mg/dL). A further retrospective cohort study in cardiovascular surgery, Görlinger et al. demonstrated that the first-line administration of the coagulation factor concentrates combined with POC testing was associated with decreased transfusion of any allogeneic blood product (52.5 vs. 42.2%; *p* < 0.0001), packed red blood cells (49.7 vs. 40.4%; *p* < 0.0001) and fresh frozen plasma (19.4 vs. 1.1%; *p* < 0.0001), whereas platelet transfusion increased (10.1 vs. 13.0%; *p* < 0.0041) [6]. These convincing results could be validated in a subsequent prospective, randomized clinical trial by Weber et al. Beside a significant lower erythrocyte transfusion rate, outcome parameters like length of intensive care unit stay, costs of hemostatic therapy and even 6-month mortality were lower in the POC treated group, too [7]. Further, in a prospective trial on major obstetric hemorrhage, Mallaiah et al. showed superior outcomes and prompt correction of the coagulation deficit by ROTEM^®^ guided administration of fibrinogen concentrate [9]. Due to the dynamics of coagulopathy, repetitive measurements are necessary. Standard coagulation parameters take 30–90 or more minutes [10] while results of viscoelastic testing can generate results within minutes [56]. There are of course limitations to the clinical applicability of viscoelastic testing. Single coagulation factor deficiencies and the effect of their substitution are not displayed specifically [57]. Additionally, detection and quantification of the effect of anticoagulants is insufficient. Platelet inhibitors can go unnoticed because of the high thrombin levels produced during viscoelastic testing. Platelets are stimulated strongest via their thrombin receptor pathway (e.g., protease-activated pathways (PAR)), which is activated by thrombin. Other pathways that are possibly blocked such as cyclooxygenase-1 (COX-1) or ADP (P2Y-12) pathways are bypassed [58].

### 4.2. Viscoelastic Parameters for Anticoagulated Patients

Considering the large number of anticoagulated patients, the administration of viscoelastic methods is nevertheless useful, e.g., atrial fibrillation occurs during the life span of 1 out of 4 individuals [59], and systemic anticoagulation with direct oral anticoagulants (DOAC’s) or vitamin K antagonists is routinely recommended for the prevention of systemic embolism [60]. As a consequence, the frequency of interventions associated with a high bleeding risk that is performed in anticoagulated patients is increasing [61]. Global coagulation assays such as PT or aPTT do not reliably and precisely detect clinically relevant DOAC levels [62]. Viscoelastic tests can indicate the presence of a DOAC. Although EXTEM CT of the ROTEM^®^ is progressively prolonged with increasing plasma concentrations of Xa or IIa inhibitors [63], their quantification requires a calibrated anti-Xa- or anti-IIa assay by the laboratory [64]. The impact of low DOAC plasma levels on ROTEM^®^ assays is poor. Nevertheless, a strong correlation of the LowTF CT between apixaban and rivaroxaban therapy with ascending drug plasma concentrations ranging from 50 to 400 ng/mL has been shown by Adelmann et al. [65]. However, to double EXTEM CT the required DOAC concentration was 1042 ± 225 ng/mL for apixaban, 134 ± 38 ng/mL for edoxaban, 176 ± 26 ng/mL for rivaroxaban and 284 ± 73 ng/mL for dabigatran. Interestingly, MA remains unchanged [63,66]. A current study of Vedovati et al. was able to accurately identify the activity of apixaban, dabigatran and rivaroxaban by ROTEM^®^ by EXTEM and ECATEM-B assay [67]. Furthermore, the parameters R time, K time, and α-angle of the TEG^®^ kaolin test were able to detect different concentrations of apixaban and dabigatran, also a higher concentration of rivaroxaban. The ACT parameter of the RapidTEG™ test of TEG^®^ was significantly and constantly different with varying concentrations of apixaban, dabigatran and rivaroxaban [68]. Bliden et al. used two TEG^®^ 6S assays (DTI and AFXa assay) to detect the anticoagulant effects of dabigatran, rivaroxaban, apixaban and edoxaban. The R-time showed a strong correlation with each [69]. The ClotPro^®^ analyzer is able to detect factor Xa antagonists using the RVV test, dabigatran using the ECA test and antifibrinolytics using the TPA test [44]. Further validation is required.

### 4.3. Algorithm-Guided POC Therapy

Correct application is crucial for successful POC diagnostics: no matter how fast and reliably a POC device generates results, it is of no use if it is not applied correctly. A predefined algorithm is therefore essential, from the complete acquisition of patient data to the subsequent targeted therapy. In summary, (A) patient-specific parameters must be generated, for which POC devices are of great use, (B) these parameters must be interpreted correctly, and very important (C) the identified abnormalities must be treated in a targeted manner. Standardization generates reproducibility, which is an advantage since it facilitates the identification of weak points in the process and promotes its continuous improvement. Several studies have shown an improved outcome of trauma patients with the generation of POC parameters and their application along with algorithms [5,70]. Such an algorithm is described and illustrated by Stein et al. [3]. These findings are supported by a Cochrane Review of Wikkelsø et al. that considered studies until 2016 on the TEG^®^ or ROTEM^®^ monitoring of hemostatic therapies as compared to standard therapy in adults and children with bleeding. Compared with transfusion policies guided by any method, a TEG^®^ or ROTEM^®^ guided management reduced overall mortality. Additionally, a significant reduction of transfused pooled red blood cells (PRBCs), fresh frozen plasma (FFP) and platelets was registered [71]. The impact of viscoelastic coagulation monitoring together with an algorithm-guided therapy has been shown in multiple meta-analyses [72,73,74,75,76,77,78].

The current COVID-19 pandemic illustrates the importance of using resources in a targeted manner. Several studies describe significant coagulation alterations in hospitalized COVID-19 patients [79,80]. POC based testing and subsequent algorithmically defined targeted therapy play a major role in the treatment of this population [81].

## 5. Platelet Function Tests

Platelets play a crucial role in hemostasis. A variety of assay methods are available for the quantitative assessment of the platelet function. Light transmission aggregometry according to Born [82] is considered the gold standard of platelet function testing. This method measures the increase of light transmission through a cuvette of platelet-rich plasma when platelets aggregate upon the addition of stimulants such as ADP, collagen, epinephrine, arachidonic acid or ristocetin. This method is labor-intensive and requires tightly controlled preanalytical conditions (e.g., manipulation and sample transport may preactivate platelets) [83]. Available POC methods have important limitations. Moreover, in patients taking antiplatelet substances the platelet-inhibitory effect observed in vitro is not necessarily equivalent to that in vivo. Nevertheless, supplementary information for coagulation management in specific situations can be obtained, e.g., the determination of interindividual variation of platelet inhibition by P2Y_12_-receptor antagonists to prevent thrombotic events after coronary stenting [84]. Regarding ROTEM^®^ platelet and Multiplate^®^, there is evidence for a predictability of postoperative blood loss (e.g., chest tube drainage) and red blood cell transfusion in patients undergoing elective cardiac surgery [85,86]. However, the impact of drug-induced platelet inhibition on early postoperative bleeding is difficult to predict and in general, the tests are not sufficiently specific [85,87]. Regarding the therapy of bleeding trauma patients, current European guidelines assume only a subordinate role, adjunct to standard laboratory [1]. Moenen et al. showed that Multiplate^®^ and PFA^®^ cannot discriminate between preoperative and referred patients with and without mild platelet function disorders (PFD’s), indicating that they are not useful as screening tests for mild PFD’s in these patients [88].

### 5.1. Tests Based on Whole Blood Aggregometry

**Figure 6:**
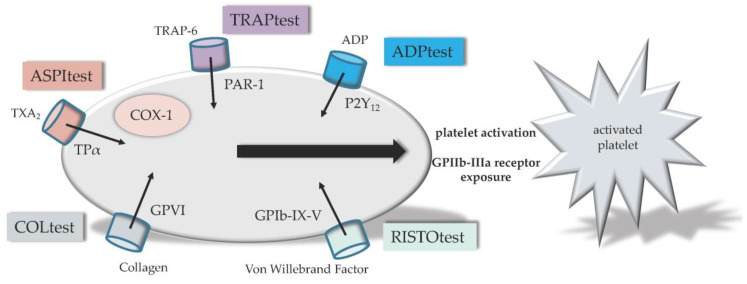
Illustration of Multiplate^®^ tests. The ADPtest detects platelets after stimulation of adenosine diphosphate (ADP) receptor pathway P2Y_12_. The TRAPtest detects platelets after stimulation with the agonist thrombin receptor activating peptide (TRAP) of the protease activated receptor-1 pathway (PAR-1). The RISTOtest detects platelets after stimulation with the agonist ristocetin of the glycoprotein Ib-IX-V (GPIb-IX-V) receptor pathway. The ASPItest detects platelets activated by arachidonic acid (AA). AA is finally converted to thromboxane A_2_ (TXA_2_) by cyclooxygenase-1 (COX-1) and others. The COLtest detects platelets activated by AA, too. Collagen is added to the sample and stimulates the collagen receptor glycoprotein VI (GPVI) pathways. No direct Glycoprotein IIb-IIIa (GPIIb-IIIa) receptor measuring.

Originally described by Cardinal and Flower 1980 for the measurement of platelet aggregation in platelet-rich plasma or whole blood [89]. The serum is stirred at 37 °C between two platinum electrodes set at a fixed distance. The position of electrodes in the cell is important, and both electrodes are facing the blood uniformly. Platelets adhere to the electrodes and cover their surface. Further adhesion of platelet aggregates depends on the addition of specific agonists, coating the electrodes and impairing the electric conduction. The magnitude of the response is proportional to the number of reacting platelets. This increase of the impedance can be displayed on any suitable chart recorder. Three principal systems are in discussion:

The Multiplate^®^ Analyzer (F. Hoffmann-La Roche AG, Roche Diagnostics, Switzerland) consists of five channels for parallel measurements. Multiple electrode aggregometry (MEA) was introduced in 2006 by Tóth et al. [90] for the measurement of platelet aggregation and platelet inhibition by aspirin or apyrase in diluted whole blood. Currently, for analysis, 300 µL of citrated whole blood is pipetted in a computer-controlled manner into the specific cups. Generation of results takes 6–10 m. Output aggregation curves describe the platelet reactivity, and an area under the curve (AUC) is determined for each assay [91]. Several assays were available and demonstrated in Figure 6. The ADPtest detects platelets after stimulation with the agonist adenosine diphosphate (ADP) of the ADP receptor pathway P2Y_12_, which is blocked by antagonists such as clopidogrel, prasugrel or ticagrelor. The TRAPtest allows the measurement of the effect of glycoprotein IIb and IIIa (GPIIb/IIIa) antagonists (e.g., abciximab, eptifibatid and tirofiban). Determining the platelet function by the agonist thrombin receptor activator peptide-6 (TRAP-6) stimulating the protease activated receptor-1 pathway (PAR-1). The RISTOtest allows the determination of platelet aggregation dependent on the von Willebrand Factor (VWF) and glycoprotein Ib (GPIb) simulated by ristocetin. Ristocetin is an agonist of the glycoprotein Ib-IX-V (GPIb-IX-V) receptor pathway. The ASPItest detects platelets activated by arachidonic acid (AA). AA is converted to prostaglandin H_2_ (PGH_2_) by cyclooxygenase-1 (COX-1), and PGH_2_ is then converted to thromboxane A_2_ (TXA_2_) by thromboxane synthase. TXA_2_ increases platelet aggregation through the TXA_2_ alpha (TPα) pathway. Non-steroidal anti-inflammatory drugs (NSAID’s) like acetylic acid are COX inhibitors. Furthermore, the COLtest detects platelets activated by AA, too. Collagen is added to the sample and binds to collagen receptors GPVI and α_2_β_1_. Stimulating this pathways leads to a release of AA [92].

The ROTEM^®^ platelet module (Instrumentation Laboratory, Bedford, MA, USA) is a set of two channels, which can be added to the standard thromboelastometric system. Handling is similar. For analysis, 300 µL of citrated whole blood is required and pipetted in a computer- controlled manner. AA (ARATEM), ADP (ADPTEM) and TRAP-6 (TRAPTEM) are used as agonists (activators) and detect the effect of COX inhibitors, ADP-receptor inhibitors or GpIIb-IIIa antagonists [93].

The TEG^®^ platelet mapping system (Haemonetics Corporation, Boston, MA, USA) is a modification of the original TEG^®^. First, a kaolin-activated test is performed to evaluate the maximal hemostatic activity. Second, a test in the presence of reptilase and factor XIIIa produces a cross-linked fibrin clot. The addition of ADP or AA stimulates differentially the role of platelet ADP or TXA2 receptors in clot formation. The effect of therapy with aspirin^®^ (AA addition) or thienopyridines (e.g., clopidogrel, ADP addition) is evaluated by comparing the TEG^®^ kaolin-activated test curve with the AA or ADP-stimulated TEG^®^ curve. Modified TEG is with the addition of a GPIIb-IIIa receptor antagonist to assess the contribution of the fibrinogen-platelet interaction to TEG parameters.

The platelet function is described by the clot formation time (ROTEM^®^) or K Index (TEG^®^). The fibrin formation is reproduced by the alpha-angle (α) in both systems. The clot formation contributed by the platelets is described by the maximum clot firmness (ROTEM^®^) or maximum amplitude (TEG^®^) [94].

### 5.2. Tests Based on Platelet Adhesion under Shear Stress

The Platelet Function Analyzer (PFA^®^, Siemens Healthineers, Munich, Germany) is based on platelets aggregating under high shear force in the presence of collagen and other platelet agonists. In vivo, this is mediated by the von Willebrand factor. The test was first described in 1995 by Kratzer et al. (Thrombostat) to investigate the effect of different platelet inhibitors such as aspirin on primary hemostasis [95]. PFA^®^ units are distributed as single use cartridges, which contain either ADP or epinephrine. A third cartridge is supplemented with P2Y_12_ and is sensitive to the effect of clopidogrel. The test is automated. Citrated whole blood is aspirated through a capillary and placed on the membrane via a small aperture of 150 μm. The high velocity of the flow generates a shear force similar to the microcirculation where VWF binds to glycoprotein Ib. The time to closure of the aperture by platelet aggregates is registered (PFA closure time), and depends on VWF, platelet reactivity, the effect of platelet inhibitors, as well as platelet count and hematocrit [94]. To a certain degree, the combination of the epinephrine and ADP units allows differentiation of severe intrinsic platelet defects (such as thrombasthenia Glanzmann or Bernard Soulier syndrome) or pronounced defects of VWF, from milder platelet function defects or the effect of antiplatelet substances such as acetylsalicylic acid [96].

### 5.3. Tests Based on Optical Detection

The VerifyNow^®^ Assay (ITC, Edison NJ, USA), formerly known as Ultegra Rapid Platelet Function Analyzer RPFA, uses a cartridge containing fibrinogen-coated beads and platelet agonists. It is a fully automated point-of-care test originally developed to monitor GPIIb-IIIa antagonists (abciximab; ReoPro) in the 1990s to prevent ischemic complications associated with unstable angina pectoris and percutaneous coronary interventions [97]. A citrated whole blood sample is inserted into a closed system using optical detection. Proportionally to the expressed GPIIb-IIIa receptors, the activated platelets bind to a fibrinogen-covered layer in the assay. TRAP (iso-TRAP) is added to analyze the inhibitory effect of intravenous platelet GPIIb-IIIa antagonists. TRAP activates the platelets, resulting in GPIIb-IIIa exposure and binding of the fibrinogen-coated beads to the platelet receptors that are not blocked. Aggregation in response to the agonist is monitored by light transmission and results (percent inhibition) are available within a few minutes [94]. There are two other cartridges available for monitoring either COX-1 (containing AA as agonist) or P2Y_12_ (containing ADP as agonist) inhibition [98].

## 6. Point-of-Care Coagulometry

PT as well as aPTT can be measured by POC coagulometers. There are several devices available. The CoaguChek^®^ (F. Hoffmann-La Roche AG, Roche Diagnostics, Switzerland) is frequently used. Its test strips include activators and a peptide substrate. Upon contact with (capillary) whole blood, the reagent (thromboplastin for PT or celite for aPTT) dissolves and initiates thrombin generation. Thrombin cleaves a synthetic peptide substrate generating an electrochemical signal, which is converted into INR or aPTT [99]. The results must be interpreted with caution since they are altered by several factors, such as coagulation factor-deficiency including fibrinogen deficiency, impaired liver function, vitamin K deficiency or consumption coagulopathy [100]. For this reason CoaguChek^®^ is approved only for the monitoring of anticoagulation with vitamin K antagonists (e.g., phenprocoumon) [101]. POC measurement of PT and aPTT are of limited value in acute bleeding, such as trauma or surgery.

## 7. Blood Gas Analysis

In principle, all blood gas analyzers are POC devices. Due to the essential physiological parameters they determine promptly, we found them worth mentioning. Arterial blood sampling allows the precise assessment of oxygenation (PaO_2_ and hemoglobin saturation), of ventilatory status (PaCO_2_) and of acid-base equilibrium (BE and pH) [102]. The parameters hemoglobin (Hb), acid-base status (pH) and calcium (Ca^2+^) are particularly important for a comprehensive coagulation management as they reflect the essential physiologic basis. Depending on the number of parameters, between 40 and 120 µL volume of whole blood is analyzed. A distinction is made between parameters which are measured (blood gases, electrolytes, CO-oximetry and metabolites) or calculated (e.g., HCO_3_^−^, BE) [103]. The spectrum of methods is ample. The parameters of blood gases (pO_2_, pCO_2_ and pH) are measured directly either electrochemically or optically. The measurement of electrolytes, especially ionized Ca^2+^, is done photometrically. Chemical sensors or fiber-optic chemical sensors are increasingly used as a standard [104]. Schober et al. [105] evaluated a portable blood gas analyzer (i-Stat 1, Abbott, Chicago, IL, USA) in prehospital helicopter medical service. Critically ill patients are managed with limited monitoring options during transport. They described benefits (e.g., portability and speed) and limitations (e.g., narrow operational temperature range).

## 8. Conclusions

Coagulation management is based on a reliable assessment of the hemostasis, which may be compromised by trauma-induced coagulopathy, of antithrombotic therapy or of other coagulopathies and during surgery. In the acute situation, POC devices are of great value as results are generated fast and reliable. Viscoelastic methods can be used for a real-time measurement of the clot formation rate and clot stability. They are of value in the early diagnosis of trauma-induced coagulopathy, for transfusion management, and a targeted hemostatic therapy within short time. Besides trauma, pathological changes can be caused by intra- and postoperative bleeding. Although platelet function plays a crucial role in hemostasis, the assessment by POC assays is unsatisfactory. Other disadvantages are the high cost of reagents, and a limited sample throughput as compared with standard laboratory assays. Additionally, an operator training is required covering the handling, test procedures, preanalytical and analytical conditions, and the interpretation of the results. Additionally, regular assay calibration is challenging.

Overall, POC-guided treatment algorithms are an essential part of coagulation management in the acute situation. The introduction of viscoelastic methods to such algorithms improves mortality, reduces the transfusion of blood components and has a cost-saving effect in the context of transfusion and coagulation support as shown in several meta-analyses. For these reasons, coagulation assays are more often integrated in multifunctional POC systems. Additionally, improved visualization of the assay results (such as Visual Clot for the ROTEM^®^) facilitates the fast and correct interpretation of results in critical situations.

## 9. Patents

- Visual Clot: U.S. Design Patent Application No. 29/725,001; Registered European Union design protection 007691399-0001 to 007691399-0012; European patent application 2019P01267EP “Method and system for monitoring a patient’s blood coagulation function.”

- Visual Patient: United States patent 10,702,214. “Method for monitoring and visualizing a patient’s medical condition”; European Union trademark 1424812 “Visual Patient”; Swiss trademark 719318 Visual Patient”; Registered European Union design protection 004064178-0001 and 004064178-0002; European patent application EP3311315A1 “Method and system for monitoring a patient’s medical condition”.

## Figures and Tables

**Table 1:** ROTEM^®^ Assays. The left column lists the main tests with the corresponding descriptions of content, activation, coagulation determinants and usage on the right column.

**EXTEM**	• Contains tissue factor (thromboplastin) as activator • Reflects the extrinsic coagulation pathway • Depends on the activity of the coagulation factors X, VII, V, II, fibrinogen and platelets
**INTEM**	• Activated by ellagic acid and phospholipids • Imitates the contact activation, reflects the intrinsic coagulation pathway • Depends on the activity of the coagulation factors XII, XI, IX, VIII, X, V, II, fibrinogen and platelets
**FIBTEM**	• Contains the platelet inhibitor cytochalasin D • EXTEM-based • Clot formation is dependent on fibrinogen concentration and fibrin polymerization independent of platelets
**APTEM**	• Contains an antifibrinolytic drug (formerly aprotinin, currently tranexamic acid) • EXTEM-based • For in vitro simulation of antifibrinolytic therapy
**HEPTEM**	• Contains heparinase • INTEM-based • To asses heparin effect

**Table 2:** TEG^®^ assays. The left column lists the main tests with the corresponding descriptions of content, activation, coagulation determinants and usage on the right column.

**RapidTEG™**	• Contains tissue factor and kaolin as activator • Reflects both, intrinsic and extrinsic coagulation pathways • Roughly analogous to activated clotting time (ACT)
**Kaolin**	• Activated by kaolin • Imitates the contact activation, reflects the intrinsic coagulation pathway
**Functional** **Fibrinogen**	• Contains tissue factor as activator and abciximab as platelet inhibitor • Clot formation is dependent on fibrinogen concentration and fibrin polymerization independent of platelets
**Native**	• Only re-calcification
**HTEG**	• Contains heparinase • Reflects the intrinsic coagulation pathway • To asses heparin effect

**Table 3:** Comparison of ROTEM^®^ and TEG^®^ parameters.

ROTEM^®^	TEG^®^	Unit	Explanation	Significance
**Clot Activation Parameters**				
Clotting Time (CT)	Reaction Time (R)	s	Time from test start to an amplitude of 2 mm	Velocity of thrombin generation
Clot Formation Time (CFT)	Kinetic Time (K)	s	Time between 2 and 20 mm clot amplitude	Kinetics of clot formation
Alpha-angle (α)	Alpha-angle (α)	degree (°)	Tangential angle at 2 mm amplitude (ROTEM^®^) or slope between R and K (TEG^®^)	Velocity of clot formation
**Clot Firmness Parameters**				
Amplitude at 5, 10 m (A5, 10)	Amplitude at 30, 60 m (A30, 60)	mm	Amplitude at set time	Clot strength
Maximum Clot Firmness (MCF)	Maximum Amplitude (MA)	mm	Greatest width achieved	Maximal cloth strength
**Clot Lysis Parameters**				
Lysis Index at 30, 60 m (LI30, 60)	Clot Lysis at 30, 60 m (CL30, 60)	%	Residual clot firmness at set time, as % of MCF	Clot stability and fibrinolysis
Maximum Lysis (ML)	unestablished	%	Maximum lysis detected during the run time, as % of MCF	Maximal clot stability and fibrinolysis
